# Comparative Assessment of Thermotolerance in Dorper and Second-Cross (Poll Dorset/Merino × Border Leicester) Lambs

**DOI:** 10.3390/ani10122441

**Published:** 2020-12-20

**Authors:** Aleena Joy, Frank R. Dunshea, Brian J. Leury, Kristy DiGiacomo, Iain J. Clarke, Minghao H. Zhang, Archana Abhijith, Richard Osei-Amponsah, Surinder S. Chauhan

**Affiliations:** 1 Faculty of Veterinary and Agricultural Sciences, The University of Melbourne, Parkville, VIC 3010, Australia; aleenajoyj@student.unimelb.edu.au (A.J.); fdunshea@unimelb.edu.au (F.R.D.); brianjl@unimelb.edu.au (B.J.L.); kristyd@unimelb.edu.au (K.D.); iain.clarke@unimelb.edu.au (I.J.C.); minghao@student.unimelb.edu.au (M.H.Z.); apayyanakkal@student.unimelb.edu.au (A.A.); ROsei-Amponsah@ug.edu.gh (R.O.-A.); 2 Faculty of Biological Sciences, The University of Leeds, Leeds LS2 9JT, UK; 3 Department of Animal Science, School of Agriculture, College of Basic and Applied Sciences, University of Ghana, Legon, Accra P.O. Box LG 226, Ghana

**Keywords:** heat stress, physiology, prolactin, sheep, thermotolerance

## Abstract

**Simple Summary:**

Selection of animal breeds that are adapted to extreme climatic conditions may help to sustain livestock production in the face of climate change. We measured the thermotolerance of 4–5-month-old Dorper and second-cross lambs (Poll Dorset × (Border Leicester × Merino)) by assessing feed intake, physiological, blood biochemical and prolactin responses. Heat stress reduced feed intake only in second-cross lambs but not in Dorpers. As expected, heat stress also increased water intake, respiration rate, rectal temperature, and skin temperature in both genotypes, but to a lesser extent in Dorpers. The comparatively lower influence of heat stress on thermotolerance indices in Dorper indicates adaptability of this breed to heat challenge.

**Abstract:**

The objective of this study was to compare the thermotolerance of second-cross (SC; Poll Dorset × Merino × Border Leicester) and Dorper lambs. Dorper and SC lambs (4–5 months of age) were subjected to cyclic heat stress (HS) (28–40 °C). The temperature was increased to 38–40 °C between 800 and 1700 h daily and maintained at 28 °C for the remainder of the day (30–60% relative humidity (RH)) in climatic chambers for 2 weeks (*n* = 12/group), with controls maintained in a thermoneutral (TN) (18–21 °C, 40–50% RH) environment (*n* = 12/group). Basal respiration rate (RR), rectal temperature (RT) and skin temperature (ST) were higher (*p* < 0.01) in SC lambs than in Dorpers. HS increased RR, RT and ST (*p* < 0.01) in both genotypes, but the levels reached during HS were lower (*p* < 0.01) in Dorpers. HS increased (*p* < 0.01) water intake to a greater extent in SC lambs, while feed intake was reduced (*p* < 0.05) by HS in SC lambs but not in Dorpers. HS increased (*p* < 0.01) blood urea nitrogen and creatinine in SC lambs only. Plasma non-esterified fatty acid concentrations were reduced (*p* < 0.05) by HS in SC lambs but increased (*p* < 0.05) in Dorpers. There was no effect of HS on pO_2_, cHCO_3_^−^ and cSO_2_, but higher (*p* < 0.01) blood pH and lower (*p* < 0.01) pCO_2_ were recorded under HS in both genotypes. Blood electrolytes and base excess were reduced (*p* < 0.01) under HS, while a genotype difference (*p* < 0.05) was only observed in blood K^+^ and hemoglobin concentrations. Basal plasma prolactin concentrations were lower (*p* < 0.01) in Dorpers but were elevated at a similar level during HS (*p* < 0.01) in both genotypes. Dorper lambs are more resilient to HS than SC lambs. Future research should focus on confirming whether the better heat tolerance of Dorpers is translated to better returns in terms of growth performance and carcass traits over the summer months.

## 1. Introduction

Increased ambient temperatures have a negative influence on ruminant production [1], ranging from mild physiological disturbances to compromised production and fertility, as well as presenting health and welfare challenges [2]. As the intensity of the heat load increases, there is a continuous and substantial decline in the productive and reproductive efficiency of livestock [3]. Animals adapt to heat stress (HS) through behavioral, physiological and metabolic responses [4], although the magnitude of these responses varies within and between breeds [5]. The ability to thermoregulate depends upon various complex interactions including anatomical and physiological factors such as morphological properties of skin and hair, sweating and respiratory capacity, endocrinological profiles, total metabolic heat production and the relationship between surface area per unit body weight or relative lung size [4,6]. Adaptive responses displayed by ruminants are reduced feed intake and initiation of respiratory and evaporative cooling mechanisms [7]. Prolonged exposure to HS, however, initiates homeostatic processes in order to cope with the stressor, including increased circulating cortisol and prolactin concentrations and lowered growth hormone and thyroid hormone concentrations [8].

Conventional genetic selection for factors such as milk yield in dairy cattle and growth rate and leanness in pigs and poultry has produced animals which produce more metabolic heat, and this is associated with lower thermotolerance [9]. Inclusion of traits associated with thermotolerance in breeding indices and greater consideration of genotype–environment interactions will identify breeds that are best adapted to specific local environments. Relatively thermotolerant genotypes have been identified and display resilience to HS in terms of productive and reproductive efficiency as well as improved adaptive responses to ensure survival [10]. On the other hand, it is evident that climate change has exposed high-producing breeds and crossbreds to temperatures beyond their comfort zones, in both their native regions as well as in other countries where they are imported for their desirable productive traits [2]. Accordingly, identification of heat-resilient breeds/crossbreds will improve productivity and fertility and welfare in the face of climate change [11].

In Australia, Merino or Merino cross sheep predominate, although other breeds such as the Dorper are gaining acceptance, due to their hardy nature, lower labor requirements and high production efficiency [12]. Dorpers are single-purpose hair sheep, tolerant of harsh environments, that were imported to Australia after 1996 and the population of this breed has increased steadily since then [12,13]. Nevertheless, second-cross (SC) Merinos still contribute 30% of the lamb meat supply. Typical SC lambs in Australia are produced following a first cross between Merino and long-wool meat breeds. Merino ewes are chosen as dams because of a plentiful supply. These ewes are good mothers and produce high-quality wool. The ram to which Merino ewes are mated can vary but is usually a long-wool meat breed such as the Border Leicester. This cross produces first-cross ewes which are generally large-framed, fertile and provide good milk. First-cross ewes are mated with a terminal sire, selected from a breed that grows fast and has strong, meat type characteristics. The ram is generally a short-wool meat breed such as the Dorset, Suffolk, White Suffolk, Southdown, Texel, Hampshire Down or Wiltshire Horn. While SC lambs are known for faster growth rates and superior carcass quality, their ability for adaptation to higher temperatures has not been studied [14]. Accordingly, the present study aimed to compare thermotolerance in Dorper and SC (Poll Dorset × (Border Leicester × Merino)) lambs constituting a crossbreed between Poll Dorset (50%), Border Leicester (25%) and Merino (25%) genetics. Thermotolerance was assessed by exposing lambs to cyclic HS over 2 weeks, with measurement of physiological and metabolic factors as well as prolactin concentrations, which are signatures of the response to HS [8,15].

## 2. Materials and Methods

### 2.1. Animals and Experimental Design

The experiment was approved by the Animal Ethics Committee (Ethics ID: 1714357.1), Faculty of Veterinary and Agricultural Sciences, The University of Melbourne, and was conducted at the Dookie Campus, Victoria, Australia (36°23′01.9′′ S 145°42′52.1′′ E).

Dorper (10 castrated males and 14 ewes) and SC lambs (8 castrated males and 16 ewes) were procured from 5 commercial breeders and used over four replications of 6 animals of each genotype. At the start of the study, the average body weights of Dorper and SC lambs were 40.9 ± 0.91 (mean ± standard error of mean (SEM)) and 42.5 ± 1.21, respectively. Following initial acclimatization to indoor housing for 1 week and individual penning for a further week, all the sheep were placed in individual metabolic cages for a further 3 days acclimatization before the experimental protocol commenced. The lambs were fed ad libitum a diet consisting of 50% commercial lamb finisher pellets (Crude protein-16% & Metabolizable energy −10.5 MJ/kg), 25% oaten chaff and 25% lucerne chaff, with free access to water. The lambs were randomly allocated to either thermoneutral (TN;18–21 °C, 30–50% RH, *n* = 12/group) or cyclic HS (28–40 °C; 40–60% RH, *n* = 12/group) for 2 weeks in climatic chambers. In the HS room, the temperature was increased to 38–40 °C between 800 and 1700 h daily and maintained at 28 °C for the remainder of the day.

### 2.2. Measurements

The temperature and humidity of climatic chambers (TN and HS) were recorded at 30-min intervals using a USB temperature/humidity data logger (TechBrands, Electus Distribution, Rydalmere, NSW, Australia) and the temperature–humidity index (THI) was calculated using the formula described by Marai et al. [16], being THI = T− {(0.31−0.0031∗RH) ∗(T−14.4)}, given T and RH are dry-bulb temperature (°C) and relative humidity (%), respectively. According to the formula, the severity of HS in lambs was categorized based on various THI ranges as follows: THI < 22.2 was considered as an absence of HS, 22.2 to 23.3 moderate HS, 23.3 to 25.6 severe HS and ≥25.6 was taken to signify extreme severe HS [17,18]. Initial and final body weights of both TN and HS animals were recorded on D1(day 1) and D14 (HS days), respectively. Feed and water intake were recorded daily by calculating the differences between feed and water offered and refused, and respiration rate (RR), rectal temperature (RT) and skin temperature (ST) were recorded thrice daily at 800, 1200 and 1600 h. Both RR and RT were recorded using standard methods described previously [19,20] and ST was measured at the right flank using a digital thermometer (Model: DT-K11A; Honsun, Shanghai, China) placed in contact with the skin until a stable reading was attained. The increment in levels of physiological parameters (RR, RT and ST) following HS exposure (at 800, 1200 and 1600 h) was calculated by subtracting individual baseline values. Blood samples (8 mL) were collected into lithium heparin vacutainers (BD, Sydney, NSW, Australia) from TN and HS animals by jugular venipuncture at 1400 h on D-1 (one day before the HS treatment), D1, D7 and D14 (HS treatment days). Blood plasma was then obtained and stored at −20 °C until assayed. Blood pH, cHgb (hemoglobin), Hct (hematocrit), glucose, lactate, creatinine, partial pressure of oxygen (pO_2_), partial pressure of carbon dioxide (pCO_2_), concentration of hydrogen carbonate (cHCO_3_^−^), anion gap, Ca^++^, Cl^−^, K^+^ Na^+^, BE b and BE ecf (base excess blood and extracellular fluid) estimation was undertaken immediately after blood collection using an Epoc analyzer (Alere Inc., Waltham, MA, USA) with version 3.29.0 system software (Sensor Configuration 33.0, Alere Inc., Waltham, MA, USA) only on day 14 as previously described [19]. Blood urea nitrogen (BUN) concentrations were estimated using a colorimetric detection kit (Thermo Fisher Scientific, Scoresby, VIC, Australia). Plasma non-esterified fatty acid (NEFA) concentrations were measured using an NEFA C kit (Wako, Novachem, Collingwood, VIC, Australia), as modified by Johnson and Peters [21] for use in 96-well plates. Inter- and intra-assay coefficients of variation were 4.8% and 6.7% for BUN, and 3.8% and 7.2% for NEFA. The ovine prolactin radioimmunoassay was performed using a primary antiserum raised in rabbits (AFPC3581069I) and ovine prolactin (NHPP, AFP10789B) as a standard for iodination [22]. The increment in prolactin concentrations of lambs following HS exposure (days D1, 7 and 14) was calculated by subtracting individual basal prolactin concentrations.

### 2.3. Statistical Analysis 

The data were analyzed using the residual maximum likelihood (REML) variance component analysis procedure for Genstat (GenStat 16th Edition; VSN International Ltd., Hemel Hempstead, UK). Fixed model effects were genotype (Dorper vs. SC), temperature (HS vs. TN), sex (castrated males vs. ewes), time (800, 1200 and 1600 h) and day (D1–D14). The random model effects were lamb and replicates, and interactions between heat treatment × genotype, treatment × time, treatment × sex, treatment × day, treatment × genotype × time and treatment × genotype × day were also tested. Multiple comparisons between the means were estimated by two-way analysis of variance (ANOVA) followed by Tukey’s post hoc test [23]. Results are presented as means ±SEM with the significance level set at *p* ≤ 0.05.

## 3. Results

### 3.1. THI, Feed Intake and Water Intake

**Figure 1:**
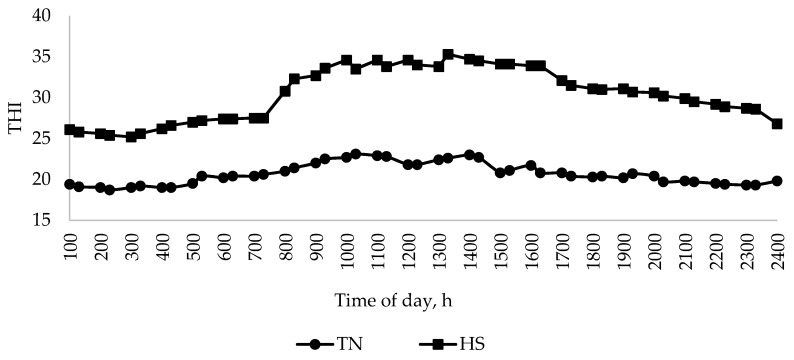
Mean temperature–humidity index (THI) over 24 h in thermoneutral (TN) and heat stress (HS) rooms, over the 14-d period of the experiment. Data are pooled for each replicate (*n* = 56 days).

The average THI for the entire experimental period (14 days) in both the TN and HS treatments is presented in Figure 1. Under TN conditions, the THI was 20.1. For the HS treatment, the THI between 800 and 1700 h was 34.4 and 26.5 for the intervening periods.

Basal feed and water intake and RT were similar in Dorper and SC lambs prior to the commencement of treatments (Table 1). RR of SC lambs was almost twice that of Dorpers (*p*< 0.01), as were prolactin concentrations (*p* < 0.01), whereas ST was 1.5 °C greater in SC (*p* < 0.05).

Initial body weights were greater (*p* < 0.01) in SC than in Dorper lambs for both TN (46.1 ± 0.34 vs. 40.8 ± 0.45 kg) and HS groups (46.3 ± 0.34 vs. 41.0 ± 0.48). However, HS reduced (*p* < 0.05) body weight in SC lambs (46.3 ± 0.34 vs. 44.5 ± 0.52) but not in Dorpers (41.0 ± 0.48 vs. 40.6 ± 0.39 kg). Feed intake was higher in SC than in Dorper lambs under TN conditions (*p* < 0.05) and was reduced by HS (*p* < 0.001; Table 2). The analysis indicated an interaction (*p* < 0.05) such that feed intake was decreased by HS in the SC lambs but not in the Dorper lambs (Table 2).

Water intake did not differ between genotypes under TN conditions and was increased by HS (*p* < 0.001; Table 2). An interaction (*p* < 0.05) between water intake and heat stress was observed between tested genotypes such that water intake was increased by HS to a greater extent in SC lambs than Dorpers (Table 2). Water intake was greater (*p* < 0.01) in ewes than castrated males (5.8 ± 0.18 vs. 5.3 ± 0.13 L/day) but there were no sex differences for any of the physiological variables, blood chemistry, feed intake or plasma prolactin concentrations (data not shown). Accordingly, the data for both sexes were pooled for further analysis.

### 3.2. Physiological Variables

RR was increased by HS (*p* < 0.001; Table 2). Further, there was an interaction (*p* < 0.001) such that RR was lower in Dorper lambs (164 breaths/min) than in SC lambs (185 breaths/min) post-HS (Table 2). RR in SC lambs was highest towards the end of the HS period (at 1600 h) (*p* < 0.01) with a shift in RR dynamics to rapid, shallow open-mouth panting. Day of treatment influenced (*p* < 0.001) RR without an interactive effect.

RT was increased by HS (*p* < 0.001). There was an interaction (*p* < 0.001) such that HS increased RT in both genotypes, but to a higher level in SC lambs than in Dorpers (Table 2). Day of treatment influenced (*p* < 0.05) RT with no interactive effects.

ST displayed significant (*p* < 0.01) genotype and treatment effects (Table 2). HS increased ST in both genotypes, reaching higher levels under HS in SC lambs, indicated by a significant (*p* < 0.01) genotype × treatment interaction. Day of treatment influenced (*p* < 0.01) ST without an interactive effect.

The incremental rise in RR levels during HS was greater (*p* < 0.001) in Dorpers than SC lambs. In contrast, the incremental rise in both RT and ST after HS exposure was greater (*p* < 0.001) in SC lambs than Dorpers (data not shown here).

### 3.3. Blood Biochemical Variables

#### 3.3.1. Blood Gases

HS reduced cTCO_2_ (*p* < 0.01), concentrations of pCO_2_ (*p* < 0.01) and cHCO_3_^−^ (*p* < 0.01) concentrations, but there were no differences between genotype (Table 3). Although there were no main effects of genotype or treatment on pO_2_ values, there was a significant (*p* < 0.05) interaction such that pO_2_ tended to be increased in HS Dorper lambs and decreased in HS SC lambs compared to TN animals (Table 3). Further, HS increased pH in both Dorper and SC lambs (*p* < 0.01; Table 3).

#### 3.3.2. Blood/Plasma Metabolite Concentrations

**Figure 2:**
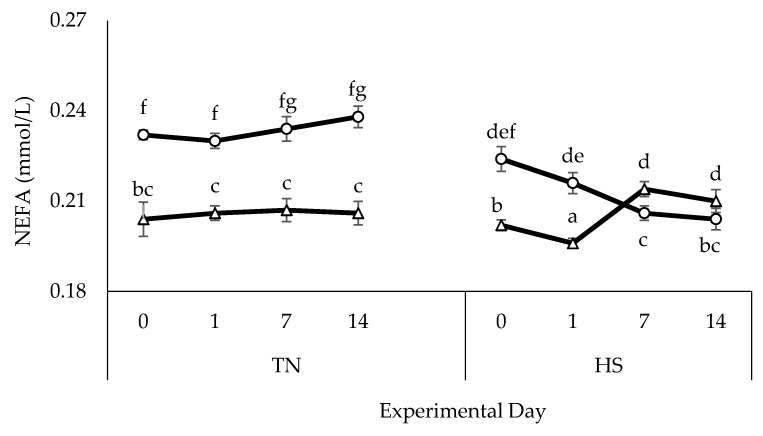
Mean (± SEM) non-esterified fatty acid (NEFA) concentrations in Dorper (∆) and SC (○) lambs subjected to either TN or HS conditions (n = 12/group). Values with different letters differ significantly for genotype (*p* < 0.001) and treatment (*p* < 0.01). Genotype × treatment and treatment × day interactions were also significantly (*p* < 0.05) different.

There were no genotype or treatment effects on blood glucose and lactate concentrations. HS caused an increase in BUN (*p* < 0.01) and creatinine (*p* < 0.01) in SC lambs only (Table 3). Under TN conditions, NEFA concentrations were greater (*p* < 0.01) in SC lambs than in Dorpers (Figure 2). There was a significant (*p* < 0.05) genotype × treatment effect for NEFA concentrations under HS with reduced concentrations in SC lambs by day 7 (*p* < 0.05) and increased (*p* < 0.05) concentrations in Dorpers compared to the respective TN animals.

#### 3.3.3. Blood Electrolyte Concentrations

In general, HS treatment affected the concentrations of most blood electrolytes except for Ca^++^, Hct and cHgb (Table 3). In both genotypes, a reduction in blood Cl^−^ (*p* < 0.05), K^+^ (*p* < 0.01), Na^+^ (*p* < 0.01), BE b (*p* < 0.01) and BE ecf (*p* < 0.01) concentrations was observed during HS. Blood anion gap concentrations were greater (*p* < 0.01) in HS animals than in TN animals. Concentrations of Hct and cHgb were greater in Dorpers (*p* < 0.05 and *p* < 0.01, respectively).

### 3.4. Plasma Prolactin Concentrations

Plasma prolactin concentrations were lower (*p* < 0.01) in Dorpers than in SC lambs under TN conditions, but values during HS were similar for the two genotypes. Exposure to HS increased plasma prolactin concentrations, irrespective of genotype (*p* < 0.01; Table 3). The incremental rise in prolactin concentrations during HS was greater (*p* < 0.01) in Dorpers than SC lambs (data not shown here).

## 4. Discussion

The thermal comfort zone for sheep has been previously reported as 12–32 °C [24], but this may differ between breeds. When subjected to HS, various thermoregulatory responses ensue but several factors including genotype, age and physiological status can influence the vulnerability of the animal. We have compared Dorper and SC lambs, showing that the former is relatively thermotolerant, based on a range of indices. Whilst it has been previously stated that Australian Merinos are relatively heat-tolerant [25], this previous study measured only basic parameters and no breed/genotype effects were compared. Others have compared Damara, Dorper and Merino ram lambs, showing that the Merinos were more susceptible to summer weight loss than the other two breeds in terms of body weight and meat quality [26].

The morphological traits of sheep are important factors of adaptability to HS, directly influencing the heat exchange rate between animals and the surrounding environment. The Dorpers used in the present study had loose white hairy wool with the head being white and free of wool, whereas SC lambs had a chalky white dense wool level to top of the muzzle. Short hair, thin skin and a lower number of hair follicles per unit area are considered to be characteristics better suited to hot environments, facilitating heat dissipation via convection [27]. Thus, it is not surprising that Dorper lambs are more heat-resilient than SC lambs, as we have demonstrated in this study. This is consistent with the findings of Titto et al. [28] (Santa Ines and Morada Nova vs. Texel, Suffolk and Ile de France), Almeida et al. [26] (Dorper, Damara vs. Merino) and Barnes and Stockman [29] (Awassi vs. Merino), who all noted that hair sheep genotypes with a loose, open fleece of hair exhibited greater thermotolerance than wool genotypes. This may be due, in part, to the observation that RR and ST are both lower in Dorpers under non-stressed conditions.

HS reduced body weight and feed intake in SC lambs but not in Dorpers. It is well known that animals under HS reduce their feed intake as an adaptive response to lower the metabolic heat production [30]. Thus, reduced feed intake in SC lambs under HS could be attributed to their greater response to the stressor, which would impact on production in this genotype [31]. Interestingly, this appears to be less of a problem in Dorpers, which maintain both body weight and feed intake under HS. This also suggests that Dorpers are more adapted to heat waves than SC lambs. Sex did not affect feed intake in the lambs in our experiment, which is in accordance with data from goats [32] and cattle [33]. HS increased water intake in both Dorpers and SC, which is consistent with other findings and is driven by dehydration, as a result of enhanced evaporative cooling mechanisms through both respiratory tracts and the skin [34,35]. Nevertheless, water intake was increased by only 27% in Dorper lambs compared to 63% in SC lambs. Thus, Dorpers are more readily adaptable to HS than SC. This is consistent with findings in cattle (*Bos indicus* vs. *Bos taurus)* [36], sheep (Awassi vs. Najdi) [37] and goats (Salem Black vs. Malabari and Osmanabadi) [7,38], where lower drinking frequency, total water intake and higher feed intake were observed in the more thermotolerant genotypes.

The magnitude of standard physiological measures of HS, such as RT, heart rate and RR, or measurements of net radiation and convection are used to evaluate HS in animals [7]. These are considered to be critical physiological indices for quantification of thermotolerance [35,39]. In the present study, both RR and ST were higher in SC lambs compared to Dorpers prior to the commencement of the HS treatment. Further, RR of both genotypes increased during HS which is in agreement with published data for a range of sheep genotypes [18,40]. As indicated above, resting RR, ST and RT were lower in Dorpers than SC lambs. Generally, animals that have higher body temperatures would be expected to direct a greater proportion of feed energy into metabolic heat production than to productivity, reducing production efficiency [41]. This suggests that efficient animals might be less susceptible to HS due to better thermoregulation [42]. However, the incremental rise in RR was greater in Dorpers compared to SC lambs. In Dorpers, the respiratory response to high heat load involved an increased breathing rate with slower and deeper panting. It has been reported that fast open-mouth panting during severe HS helps to reduce metabolic heat production and also represents an increase in susceptibility to heat load [43]. HS increased RT irrespective of genotype, indicating that thermoregulatory responses were insufficient to maintain thermal equilibrium. The fact that SC lambs had higher RT than Dorpers also indicates the better adaptive capability of the latter. Additionally, the production of heat due to feed intake had only a slight impact on RT in lambs because the increased temperature under HS was sustained in SC in spite of reduced feed intake. Dorpers did not show any reduction in voluntary feed intake while maintaining lower RT. This indicates that Dorpers are less susceptible to HS than SC as a consequence due to their superior thermoregulation. Skin temperature of lambs was elevated during HS, possibly due to enhanced blood flow to the skin, facilitating heat dissipation [39]. These data are consistent with those of Chauhan et al. [18] and DiGiacomo et al. [20], but the higher absolute values and greater incremental rise for ST and RT in SC than in Dorper lambs during HS provides further evidence that the latter are more thermotolerant. While Butswat et al. [44] reported differences between males and females for all physiological coefficients (RR, RT and HR) in the Yankassa sheep breed, the similarity between the sexes in the present study could be due to the males being castrated.

Increased RR during severe HS may result in respiratory alkalosis in sheep [45], greater heat dissipation and increased alveolar ventilation. An associated elevation in CO_2_ excretion shifts the bicarbonate equilibrium to H_2_CO_3_ from H^+^ and HCO_3_^−^ [46]. In the present study, we observed a reduction in pCO_2_ and cHCO_3_^-^ in both Dorper and SC lambs after HS exposure. In addition, HS increased blood pH values in both genotypes. Lower pCO_2,_ HCO_3_^−^ and actual base excess resulting in increased HCO_3_^−^ excretion from the kidney may lead to higher blood pH in ruminants [19]. A different pattern was seen in pO_2_ with opposite trends in Dorper (increase) and SC (decrease) lambs. A similar pattern for blood oxygen saturation was also reported in Omani and Merino sheep breeds, being increased in Omani and reduced in Merinos after HS exposure [46]. The decreasing oxygen concentrations in SC genotypes are indicative of faster, shallow respiration patterns in SC lambs than in Dorpers; hence, SC lambs were not able to saturate an adequate amount of oxygen into the blood by removing CO_2_ under HS.

Blood glucose and lactate concentrations did not change under HS, consistent with other data in sheep and goats during summer [39,47]. Usually, an inadequate feed intake or increase in circulating basal insulin concentrations leads to lower glucose concentrations in ruminants after chronic HS [48,49]. However, the SC lambs in our study showed no reduction in blood glucose concentration after a relatively mild reduction in voluntary feed intake; this could be an adaptive response to ensure supplementary glucose supply through the mobilization of adipose stores or hepatic gluconeogenesis. HS increased BUN and creatinine concentrations in SC lambs only, again indicating high susceptibility of this genotype to HS. A similar result of a high BUN level in the thermo-susceptible breed (Merino) was also reported by Srikandakumar et al. [46]. The higher BUN concentrations in SC lambs during HS may reflect lower renal blood flow, as a result of redistributed blood flow to the skin surface for enhanced heat dissipation [50]. In addition, a heat-induced increase in BUN could be the result of muscle breakdown, being a byproduct of gluconeogenesis during amino acid catabolism [48,51]. Wheelock et al. [52] interpreted higher circulating BUN concentrations as being due to the inefficient rumen nitrogen metabolism converting rumen ammonia to microbial protein. Elevated creatinine concentrations in SC lambs also indicated their lower thermotolerance, suggesting a change in protein metabolism shifting towards catabolism during HS. The higher energy requirement required to maintain homeothermy in this genotype would have been met by enhanced tissue protein catabolism, which leads to increased creatinine concentrations [53]. Creatinine is also an indicator of kidney function among nitrogenous substances in the blood [54]. Dorper lambs can maintain baseline creatinine concentrations, perhaps indicating normal kidney function of this genotype during HS. Plasma NEFA concentrations fell slightly during HS in SC lambs but increased markedly in Dorpers. Most of the HS studies conducted in cattle [52], pigs [55] and sheep [50] have indicated a little change in NEFA during HS despite a reduction in feed intake. It could be speculated that reduced plasma NEFA concentrations in SC lambs (despite reduced feed intake) during HS are an adaptive survival response to increased heat load, as β-oxidation of NEFA produces more metabolic heat than carbohydrates [52]. Sano et al. [56] also reported reduced NEFA concentrations in heat-stressed Corriedale ewes. Plasma NEFA concentrations are highly correlated with NEFA entry rate and fat mobilization in small ruminants [57]. Elevated NEFA concentrations by HS in Dorpers could be a metabolic response to support the higher energy demand for thermoregulatory responses and is consistent with them being better adapted to HS. Likewise, Sevi et al. [58] and Mahjoubi et al. [59] also demonstrated increased circulating NEFA concentrations in heat-stressed Comisana and Afshari sheep breeds.

Prolactin is a multi-functional hormone involved in a broad variety of biological actions related to reproduction [60,61], osmoregulation [15], metabolism [62], immune function [63,64], behavior [65] and sweating [66,67] in ruminants. Plasma prolactin concentrations increase during HS in ruminants and are indicative of the possible modulatory role of prolactin in an adaptive response to hot environments including effects on thermoregulation [8], water and electrolyte balance [15,68], immune function [69] and sweating [67]. Higher prolactin concentrations also affect water conservation, reducing renal fluid and electrolyte excretion during HS [68]. Prolactin receptors (PRLR) in ovine sweat glands suggest a role for prolactin to increase sweating during HS [70]. Comparative studies between slick and wild genotypes in cattle indicate that a PRLR mutation may confer supplementary thermotolerance to cattle beyond its influences on short hair length and also suggested a greater HS response in SLICK genotypes with lower RT, RR and ST and enhanced sweating [67,71]. SLICK animals have shorter hair and lower hair follicle density than wild genotypes and are associated with a frameshift deletion within the last exon of PRLR that shortens a portion of the cytoplasmic domain of the protein. Salah et al. [72] demonstrated a significant reduction in voluntary feed intake and increased body temperature in heat-stressed lambs injected with bromocryptine (used as a prolactin secretion suppressor), further implicating a role for prolactin in response to HS. Similar results were also observed in goats such that suppression of prolactin concentrations produced severe hyperthermia during the hottest part of the day [73]. This suggests that the elevated prolactin concentration is an adaptive response to limit the rise in body temperature during HS. While the concentrations of prolactin reached under HS were similar in both genotypes, the incremental rise was greater in Dorpers, due to their lower baseline concentrations. The modulatory role for prolactin in thermoregulation and the relationship between the magnitude of the rise in concentrations and the degree of thermotolerance require further investigation.

## 5. Conclusions

The present study provides further insight into the comparative thermotolerance of Dorper and SC lambs. Significant genotype differences in the response to HS were evident in a number of parameters, namely, water intake, feed intake, RR, RT, ST, BUN, creatinine, NEFA and prolactin concentrations. This study confirms that Dorpers are more thermotolerant than SC lambs constituting a crossbreed between Poll Dorset (50%), Border Leicester (25%) and Merino (25%) genetics. Further research to determine whether the relative heat tolerance of Dorpers is translated to superior growth and carcass traits during the summer months is warranted. Thus, breeding more heat-tolerant lambs, such as Dorper, rather than SC lambs represents a potentially viable strategy to sustain sheep production under climate change impacts on livestock.

## Figures and Tables

**Table 1:** Mean (±SED) physiological parameters in Dorper and second-cross (SC) lambs prior to the imposition of thermoneutral (TN) and heat stress (HS) treatments (*n* = 24/genotype).

Variables	Genotype		Pooled SED	Significance (*p*-Value)
	Dorper	Second-Cross		
Water Intake (L)	5.3	5.5	0.54	NS
Feed Intake (kg)	1.29	1.31	0.07	NS
Respiration Rate (breaths/min)	52.5	91.3	5.92	<0.01
Rectal Temperature (°C)	39.28	39.34	0.10	NS
Skin Temperature (°C)	36.2	37.7	0.21	0.04
Prolactin Concentration (ng/mL)	75.5	158.0	18.41	<0.01

NS—non-significant, SED—standard error of differences.

**Table 2:** Mean (±SED) physiological parameters in Dorper and SC (pooled across the experimental period of 14 days) lambs subjected to either TN or HS conditions (*n* = 12/group).

Variables	Dorper		Second-Cross		Pooled SED	Significance (p-Value)			
	TN	HS	TN	HS		Genotype	Trt	Day	Genotype * Trt
Feed Intake (kg)	1.3 ^b^	1.3 ^b^	1.4 ^c^	1.2 ^a^	0.02	<0.05	<0.01	NS	<0.01
Water Intake (L)	4.7 ^a^	6.0 ^b^	4.8 ^a^	7.8 ^c^	0.2	<0.01	<0.01	NS	<0.01
Respiration Rate (breaths/min)	74 ^a^	164 ^c^	108 ^b^	185 ^d^	1.9	<0.01	<0.01	<0.01	<0.01
Rectal Temperature (°C)	39.24 ^a^	40.22 ^c^	39.68 ^b^	40.51 ^d^	0.02	<0.01	<0.01	<0.01	<0.01
Skin Temperature (°C)	36.71 ^a^	38.6 ^c^	37.86 ^b^	39.48 ^d^	0.05	<0.01	<0.01	<0.01	<0.01

TN—thermoneutral, HS—heat stress, Trt—treatment, SED—standard error of differences. * indicates interaction between genotype and treatment, Values bearing different superscripts within the row on the respective mean values differ at *p* < 0.05

**Table 3:** Mean (±SED) values for blood gases, metabolites and electrolytes in blood in Dorper and SC lambs under TN or HS conditions for 14 days (*n* = 12/group).

Variables	Dorper		Second-Cross		Pooled SED	Significance (*p*-Value)		
	TN	HS	TN	HS		Genotype	Trt	Genotype * Trt
Blood Gases								
pCO_2_ (mmHg)	37.29 ^b^	29.11 ^a^	39.93 ^b^	27.38 ^a^	1.27	NS	<0.001	NS
pO_2_ (mmHg)	34.64 ^a^	37.60 ^a^	40.83 ^a^	35.61 ^a^	0.07	NS	0.09	0.02
cHCO_3_^−^ (mmol/L)	26.88 ^b^	23.18 ^a^	28.24 ^b^	21.29 ^a^	0.68	NS	<0.01	NS
cTCO_2_	29.44 ^b^	22.01 ^a^	28.03 ^b^	22.12 ^a^	1.02	NS	<0.001	NS
Ph	7.46 ^ab^	7.50 ^c^	7.47 ^ab^	7.51 ^c^	0.01	NS	<0.001	NS
Blood/Plasma Metabolite Concentrations								
Glucose (mmol/L)	4.62 ^a^	4.54 ^a^	4.33 ^a^	4.35 ^a^	0.14	0.09	NS	NS
Lactate (mmol/L)	0.83 ^a^	1.07 ^a^	0.92 ^a^	0.82 ^a^	0.15	NS	NS	NS
BUN (mg/dL)	18.21 ^a^	19.18 ^a^	19.20 ^a^	21.93 ^b^	1.12	0.02	<0.01	NS
Creatinine (mg/dL)	0.85 ^a^	0.89 ^a^	0.90 ^a^	1.03 ^b^	0.03	<0.001	<0.01	0.09
Blood Electrolyte Concentrations								
Ca^++^ (mmol/L)	1.37 ^a^	1.34 ^a^	1.36 ^a^	1.35 ^a^	0.02	NS	NS	NS
Cl^−^ (mmol/L)	109.5 ^b^	107.7 ^a^	109.4 ^b^	108.3 ^a^	0.62	NS	0.03	NS
K+ (mmol/L)	4.79 ^c^	4.43 ^ab^	4.68 ^bc^	4.26 ^a^	0.13	NS	<0.01	NS
Na+ (mmol/L)	148.1 ^a^	143.1 ^b^	148.5 ^a^	143.6 ^b^	1.02	NS	<0.001	NS
cHgb (g/dL)	8.93 ^a^	8.74 ^a^	8.15 ^a^	8.24 ^a^	0.21	0.04	NS	NS
BE b (mmol/L)	4.03 ^b^	−1.13 ^a^	3.03 ^b^	−1.06 ^a^	0.82	NS	<0.001	NS
BE ecf (mmol/L)	4.35 ^b^	−1.84 ^a^	3.18 ^b^	−1.81 ^a^	1.06	NS	<0.001	NS
AGapK	15.17 ^a^	18.92 ^b^	16.92 ^ab^	18.33 ^b^	0.79	NS	<0.01	NS
Hct	26.33 ^b^	25.75 ^ab^	24.01 ^a^	24.17 ^ab^	0.86	0.02	NS	NS
Plasma Hormone Concentrations								
Prolactin Concentration (ng/mL)	73.4 ^a^	219.2 ^c^	153.6 ^b^	220.7 ^c^	30.42	0.03	<0.001	0.004

TN—thermoneutral, HS—heat stress, Trt—treatment, SED—standard error of differences, NS—non-significant, pCO_2_—partial pressure of carbon dioxide, pO_2_—partial pressure of oxygen, cHCO_3_^−^—concentration of hydrogen carbonate, BUN—blood urea nitrogen, cHgb—hemoglobin, BE b—base excess blood, BE ecf—base excess extra cellular fluid, AGapK—anion gap, Hct—hematocrit. * indicates interaction between genotype and treatment, Values bearing different superscripts within the row on the respective mean values differ at *p* < 0.05.
